# Knowledge Translation as Cultural and Epistemic Translation: Comment on "Sustaining Knowledge Translation Practices: A Critical Interpretative Synthesis"

**DOI:** 10.34172/ijhpm.2023.7873

**Published:** 2023-03-13

**Authors:** John Ødemark

**Affiliations:** Department of Culture Studies and Oriental Languages, University of Oslo, Oslo, Norway

**Keywords:** Knowledge Translation, Actor Network Theory, Sociology of Translation, Cultural Translation, Epistemology

## Abstract

This commentary examines the claim made by Borst et al that knowledge translation (KT) should look to Science and Technology Studies (STS), the sociology of translation, and constructionist views on knowledge, and begin to think of the sustainability of a certain practice as construction work in continuous progress, and not as states to be reached once and for all. While endorsing this claim, the present commentary also argues that what it calls the "epistemic reframing" behind the new construal of KT in Borst must be supplemented with approaches that goes beyond the sociology of translation. The commentary claims that this epistemic shift hinges upon a shift in the narrative framing of KT, and that we need to consider the broader narrative and historical ideology of knowledge dissemination behind KT, and that a failure to do so, leaves us with KT seen as a linear transmission of "true" knowledge to peoples and places lacking such knowledge.

## Introducing the Sociology of Translation for Knowledge Translation

 As knowledge translation (KT) and some notion of “sustainability” is increasingly articulated in the literature, “*Sustaining Knowledge Translation Practices: A Critical Interpretative Synthesis” *is a timely and important contribution. The article is well written and intellectually coherent, and the design of the literary review and the critical interpretative synthesis (CIS) seems to be sound. Moreover, Borst et al also offer an easily accessible introduction to Science and Technology Studies (STS) and Actor Network Theory for scholars and health professionals without any prior knowledge of STS (or the wider sociology and history of science of which STS forms a part). In fact, the condensed presentation of insights from STS is impressive in its pedagogical economy — and clarity.

 At the outset Borst et al sketch the background of their study: “*The health policy and systems research literature increasingly observes that knowledge translation (KT) practices are difficult to sustain. An important issue is that it remains unclear what sustainability of KT practices means and how it can be improved. The aim of this study was thus to identify and explain those processes, activities, and efforts in the literature that facilitate the sustaining of KT practices in health policy-making processes.*”^1^ What, then, is the sustainability of KT practices, and how can this (apparently still enigmatic process) be improved?

 The take home message of the article is that KT should turn to STS and the notion of translation developed there, often referred to as *the sociology of translation*, to understand and facilitate the sustainability of KT practices.This is so, the authors claim, because STS is both *constructivist* in its epistemology — in contrast to *positivist* KT — and has developed notions of translation that pinpoint the processual aspect of all knowledge practices: “*In French, translation connotes both transformation and displacement. Within STS, this emphasis on transformation and displacement is used to describe how networks of actors are made, and often changed, in the process of knowledge production and utilisation*” (p. 5). The French dictionary and STS, then, converge around the idea that translation, science, and all kinds of knowledge practices inevitably encompasses “transformation” and “displacement.”Accordingly, KT should look to STS and a constructionist view on knowledge, and through what I will call an “epistemic reframing,” begin to think of the sustainability of a certain practice as construction work in continuous progress — and we may add, citing B. Latour, as the continuous* care* for all the human and non-human things that support the practice in question and the science/s it translates.^2^

 It is my contention, then, that the sociology of translation is a good place to start formulating a more processual form of KT. However, I also claim that we will need to go beyond the sociology of translation to revision KT. In the following, I will begin with a few general thoughts about KT as I understand it, through “contexting” it in my own and colleagues’ work on KT as cultural translation. Next, I will return, in a slightly more critical vein than above to Borst et al before I finally relate the deployment of STS in KT to what I called “epistemic reframing” above. This epistemic “gestalt” shift — seeing KT and sustainability as a continuous process of construction and not as an accomplished state — hinges upon a shift in the narrative framing of KT, I maintain.Moreover, I also believe that we should consider the broader narrative and historical ideology of knowledge production and knowledge dissemination behind KT, and that a failure to do so, leaves us with KT seen as a linear transmission of “real” and “true” knowledge to peoples and places lacking such knowledge.

## Reframing the Epistemic Ideology Behind Knowledge Translation—or Science as Culture

 KT was devised as an answer to a problem of governance and efficacy; the need to *implement *new, scientifically warranted knowledge in the world. The assumption was — and often is — that relevant, biomedical knowledge already exists, but that there is a knowledge* cleft* between state-of-the-art scientific knowledge and the wider world. Hence, the all-important task for KT is to reduce the gap between theory and practice by making medical practice knowledge based. WHO’s definition of KT is a clear case in point: “*Knowledge translation (KT) has emerged as a paradigm to address many of the challenges and start closing the ‘know-do’ gap. KT is defined as ‘The synthesis, exchange, and application of knowledge by relevant stakeholders to accelerate the benefits of global and local innovation in strengthening health systems and improving people’s health.’”*^3^

 The space that KT is supposed to bridge is the one between science and social practice, and the objective is to close the ‘know-do gap,’ ie, a distance figured as an epistemological space between theory and practice. While inter-lingual translation crosses a boundary between languages, KT aims to cross the space between biomedical science and practical healthcare. Ideally, there should be an *equivalence *of some sort between the message produced by science (theory) and its application in practice.In other words, the objective of KT as a form of translation is to bridge the gap between knowing and doing, and thus reduce the distance between these poles by transporting knowledge, in a linear way, from a position characterized as epistemic* plenitude* to one characterized by epistemic* lack*.

 This view of KT is deeply informed by the narrative assumption, or metanarrative, of modernity celebrating the rise of reason and the rational subject freed from “superstition” and local belief.^4^ KT is based on an enlightenment model of knowledge dissemination; knowledge should trickle down from (elite) theory into (popular) practice, and thus substitute a lack of knowledge with state-of-the-art science. Translational shifts are unwarranted since knowledge has already reached its culmination in the scientific “source text.” Indeed, this is also an uncritical transfer of an ideology that sees (linguistic) translation as a practice aiming at equivalences between a source text and a target text, as governed by the norm of fidelity to the source – and construes the translator’s work, and hence the translational chain, as ‘invisible.’^5^

 Bearing this in mind, Greenhalgh et al diagnosed KT as epistemologically naïve, and claimed that the time had come to “drop the knowledge translation metaphor.”^6^ While wholly in line with this epistemological criticism of KT, others have asserted that an expanded notion of translation might help us devise forms of KT more attuned to biological, epistemological, and cultural complexities that the transfer of science inevitably will run into.^7,8^ In a recent World Health Organization (WHO) report on KT and cultural contexts, these positions converge. It is asserted that the “*approach to KT as a kind of cultural translation is based on a complex whole definition of culture as informing all kinds of knowledge. Concepts of translation in a cultural and material sense see translation as not only communicating knowledge but also creating knowledge. The implication of such a broad understanding of translation is that the distinction between science and its translation (inherent in many KT models, for example concepts such as the know–do gap) is impossible and unproductive to maintain. This needs to be taken into consideration, and tools and mechanisms are required that can guide policy-makers*.”^9^ Here, then, it is argued that KT will benefit from (*i*) acknowledging its own cultural positionality; it is itself a kind of cultural translation as it moves insight from one epistemic culture (biomedicine) to other social places and epistemic cultures, often with competing truth claims — and (*ii*) from incorporating more theoretical notions of translation developed in the human sciences and pinpointing translation as a broadly conceived cultural process. How does such a view compared with the claims made by Borst et al, and a KT based upon the sociology of translation?

## Supplementing Knowledge Translation With the Sociology of Translation

 On the one hand, Borst et al clearly follow a critical trajectory like the one mapped above; they acknowledge the creativity and productivity of translation (to be sure, this an axiomatic assumption in STS). Consequently, they are also sceptical of seeing the translations process as one of inserting an intact scientific “message” in a “foreign cultural context.” On the other hand, the integrity of KT as a practice that needs to be *sustained* by devices *external* to the translated message (science, biomedicine) does not appear to be questioned. The same applies to the idea of KT as basically a *linear* process that should be supplemented — along the way — with tools and instruments of translation from STS. If the tools of translation gets more sophisticated, the directionality of translation remains the same, unilinear. This is clear from how the “compass question” is formulated: “*which insights from the STS literature can help in better understanding how KT practices in the health policy- and health systems sector can be sustained?”* (p. 3). STS, then, is here construed as something that can be *added on* to KT to better “sustain” knowledge already produced in a process that is still essentially linear.

 In contrast to the contributions cited in the previous section, which explores broad humanistic, cultural, and text-oriented views of translation, Borst et al economically and consistently stick to how STS construe translation. Indeed, this is one of the strengths of their work, but it also raises certain intriguing questions about methods such as the CIS review, and interpretative/hermeneutic/narrative literature reviews in general, and how these can be used to translate (!) between qualitative and quantitative research, and what Borst et al calls different epistemologies: “*The STS literature was included as to enrich the review with constructivist social scientific perspectives on sustainability and KT*.” One wonders, though, if the sociology of translation is really a good instrument to use in the translation between, and (interpretative) synthesis of, qualitative and quantitative evidence, and the natural and human sciences. Callon, for instance, in a seminal paper called upon by the authors, also aimed to develop a *symmetrical *perspective that used the same language of description on human and non-human actors (scientist and scallops in his case), and hence, in the name of symmetry (much like positivism itself) abolished hermeneutics and culture from the analytical equation (the action of scientist and scallops should be described in the same language and explained in the same way).^10^ Hence, if we take the postulate of *generalized *symmetry seriously, there will be no need for translating between the qualitative and the quantitative, causes and meanings. In contrast, proponents of KT as a kind of cultural translation have argued for seeing KT as a bidirectional exploration of both “*biomedicine (avoiding simplistic reductions of life to biology) and the humanities (shunning simplistic reductions of suffering and health injustice to cultural relativism)*.”^11^ Thus exploring the asymmetries and tensions between medicine and the humanities.

 Although the turn to the sociology of translation to supplement the understanding of sustainability and translation in KT is productive and rewarding, then, it also raises methodological and philosophical questions. Borst et al make two distinct steps in their innovative calibration of the sociology of translation and KT. Firstly, we have what I above called an epistemic reframing of KT. This epistemic reframing, which makes and marks the shift from seeing “sustainability” as an end-state to a continuous process, obviously has consequences for the understanding of KT and for practical KT work. The latter practical aspects take us to what I will call a *methodological* level, which gives us an STS-influenced vocabulary for describing the KT process: “*The synthesising argument of this CIS is that conceptualisations of sustainability of KT practices would benefit from a shift from viewing sustainability as an end-state towards sustaining as the (often mundane) work that is required to make and keep KT practices productive. In the literature we noticed that this sustaining work can be divided into three work processes. Our proposition is that these processes of translating, contexting, and institutionalising together can both explain and guide the sustaining of KT practices*” (p. 8).

 This sequencing of the KT process in three different stages is innovative and potentially rewarding. In this new vocabulary, “*translating*” refers to the actor, networks and further webs of connections that transport knowledge – as well as the “mere” transfer of the content of knowledge, like “science.” “*Contexting*” refers to the way KT actors can — actively — construct contexts that keep the connections that enable translation productive, while “*institutionalizing*” is used to label the deployment of institutions to establish a temporary basis and social durability for KT practices (p. 8). These are processual labels, then, for implementing and sustainingthe translation of a KT practice (STS scholars are fond of *verbing*), the two last naming processes of continuous attachment between different KT actors and practices and their environment. Her as well it appears that the narrative model of linear knowledge dissemination from science to culture is maintained; “translating,” “contexting,” and “institutionalizing” seem to be called upon to reproduce (or sustain!) activities meant to increase the control science and knowledge already formed can take over a new domain.

## Epistemological Reframing and Narrative Assumptions

 Certainly, KT should look to STS and constructionist views on knowledge and — through what I have called an “epistemic reframing” — begin to think of the sustainability of a certain practice as construction work in continuous progress. It is easy to agree, and it is easy to welcome empirical studies and new practices based upon the new and innovate grid established by Borst et al (“translating,” “contexting,” and “institutionalizing”). KT as a kind of sociology of translation, however, still seem to be informed by a master narrative of enlightenment and modernity, a narrative assumption where translation moves from a position characterized as epistemic* plenitude* to one characterized by epistemic* lack*, and not between (often competing) epistemic cultures where both facts and values are regularly contested. Hence, it is not always easy to ascertain what knowledge or science one should support and sustain. A case in point is masking during the COVID-19 pandemic, which was contested politically but also raised questions in different disciplines and sciences about what constituted evidence and knowledge, ie, a struggle between different epistemic cultures not univocal science and “culture.”

 In conclusion, the reframing of KT as a translation process performed by Borst et al also demonstrate the need for going beyond STS and the sociology of translation by reflecting further upon the narrative framings of processes, like the idea if sustaining certain forms of knowledge: Neither “knowledge” nor “sustainability” is a state to be reached as some kind of final, narrative culmination, as the happy ending of fairy tales – or pedagogical theories and philosophies of modernity pinpointing the rise of reason and the rational subject freed from “superstition” and local cultures.

## Ethical issues

 Not applicable.

## Competing interests

 Author declares that he has no competing interests.

## Author’s contribution

 JØ is the single author of the paper.
